# Combination of serum and peritoneal 1.3-beta-d-glucan can rule out intra-abdominal candidiasis in surgical critically ill patients: a multicenter prospective study

**DOI:** 10.1186/s13054-023-04761-7

**Published:** 2023-11-30

**Authors:** Emmanuel Novy, Jérémie Rivière, Maxime Nguyen, Gaëlle Arfeuille, Guillaume Louis, Bélaïd Bouhemad, Julien Pottecher, Stéphane Hecketsweiler, Adeline Germain, François-Xavier Laithier, Marie-Reine Losser, Anne Debourgogne, Yohann Bernard, Hélène Rousseau, Cédric Baumann, Amandine Luc, Julien Birckener, Marie-Claire Machouart, Philippe Guerci

**Affiliations:** 1 Service d’Anesthésie-Réanimation et Médecine Péri-opératoire, CHRU Nancy – Hôpitaux de Brabois, 54500 Vandœuvre-Lès-Nancy, France; 2 SIMPA, UR7300, Université de Lorraine, 54500 Vandœuvre-Lès-Nancy, France; 3 Service de Réanimation Polyvalente, CHR Metz-Thionville, 57000 Metz, France; 4 Service d’Anesthésie-Réanimation et Médecine Péri-Opératoire, CHU Dijon, 21000 Dijon, France; 5 INSERM UMR1231, Université de Bourgogne-Franche Comté, 21000 Dijon, France; 6 Service d’Anesthésie-Réanimation et Médecine Péri-Opératoire, Hôpital de Hautepierre, Hôpitaux Universitaires de Strasbourg, 67200 Strasbourg, France; 7 UR3072, FMTS, Faculté de Médecine, Maïeutique et Science de la sante, Université de Strasbourg, 67000 Strasbourg, France; 8 Service de chirurgie digestive, CHRU Nancy – Hôpitaux de Brabois, 54500 Vandœuvre-Lès-Nancy, France; 9 NGERE, U1256, Université de Lorraine, 54500 Vandœuvre-Lès-Nancy, France; 10 DCAC, INSERM 1116, Université de Lorraine, 54500 Vandœuvre-Lès-Nancy, France; 11 Service de mycologie et parasitologie, CHRU Nancy – Hôpitaux de Brabois, 54500 Vandœuvre-Lès-Nancy, France; 12 Délégation À la recherche et à l’innovation, CHRU de Nancy, 54500 Vandœuvre-Lès-Nancy, France; 13 Unité de Méthodologie, data management et statistiques, DRCI, CHRU de Nancy – Hôpitaux de Brabois, 54500 Vandœuvre-Lès-Nancy, France

**Keywords:** Intra-abdominal candidiasis, *Candida*, Beta-d-glucan, Diagnostic, Critically ill patient

## Abstract

**Background:**

Intra-abdominal candidiasis (IAC) is difficult to predict in critically ill patients with intra-abdominal infection, leading to the overuse of antifungal treatments. Serum and peritoneal 1.3-beta-d-glucan (sBDG and pBDG) have been proposed to confirm or invalidate the diagnosis of IAC, but clinical studies have reported inconsistent results, notably because of heterogeneous populations with a low IAC prevalence. This study aimed to identify a high-risk IAC population and evaluate pBDG and sBDG in diagnosing IAC.

**Methods:**

This prospective multicenter noninterventional French study included consecutive critically ill patients undergoing abdominal surgery for abdominal sepsis. The primary objective was to establish the IAC prevalence. The secondary objective was to explore whether sBDG and pBDG could be used to diagnose IAC. Wako^®^ beta-glucan test (WT, Fujifilm Wako Chemicals Europe, Neuss, Germany) was used for pBDG measurements. WT and Fungitell^®^ beta-d-glucan assay (FA, Associate of Cape Cod, East Falmouth, USA) were used for sBDG measurements.

**Results:**

Between 1 January 2020 and 31 December 2022, 199 patients were included. Patients were predominantly male (63%), with a median age of 66 [54–72] years. The IAC prevalence was 44% (87/199). The main IAC type was secondary peritonitis. Septic shock occurred in 63% of cases. After multivariate analysis, a nosocomial origin was associated with more IAC cases (*P* = 0.0399). The median pBDG level was significantly elevated in IAC (448 [107.5–1578.0] pg/ml) compared to non-IAC patients (133 [16.0–831.0] pg/ml), *P* = 0.0021. For a pBDG threshold of 45 pg/ml, the negative predictive value in assessing IAC was 82.3%. The median sBDG level with WT (n = 42) at day 1 was higher in IAC (5 [3.0–9.0] pg/ml) than in non-IAC patients (3 [3.0–3.0] pg/ml), *P* = 0.012*.* Similarly*,* median sBDG level with FA (n = 140) at day 1 was higher in IAC (104 [38.0–211.0] pg/ml) than in non-IAC patients (50 [23.0–141.0] pg/ml), *P* = 0.009*.* Combining a peritonitis score < 3, sBDG < 3.3 pg/ml (WT) and pBDG < 45 pg/ml (WT) yielded a negative predictive value of 100%.

**Conclusion:**

In critically ill patients with intra-abdominal infection requiring surgery, the IAC prevalence was 44%. Combining low sBDG and pBDG with a low peritonitis score effectively excluded IAC and could limit unnecessary antifungal agent exposure.

*Trial registration*: The study was registered with ClinicalTrials.gov (ID number 03997929, first registered on June 24, 2019).

**Graphical abstract:**

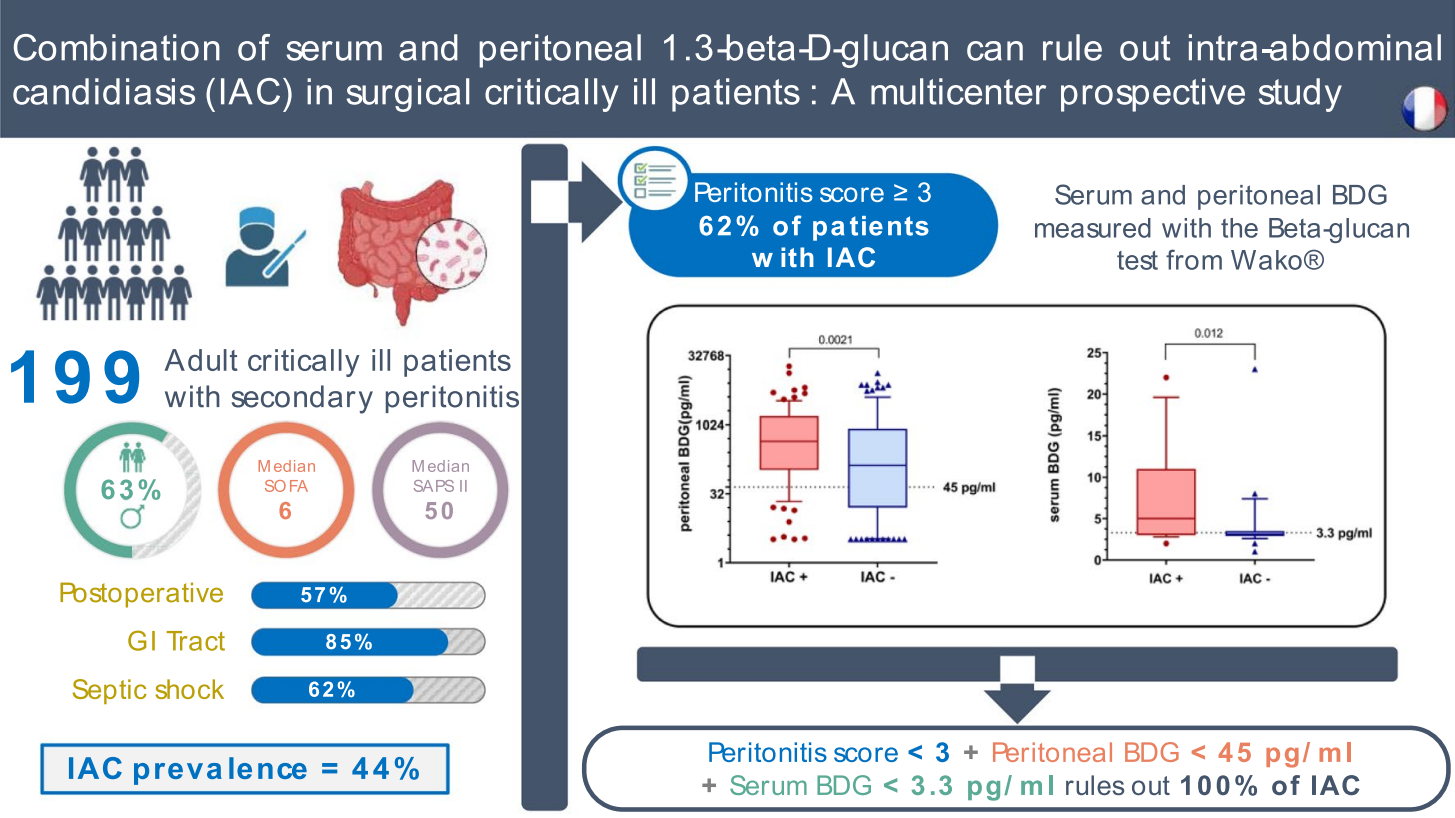

**Supplementary Information:**

The online version contains supplementary material available at 10.1186/s13054-023-04761-7.

## Take-home message

- Critically ill patients with nosocomial secondary peritonitis and a peritonitis score ≥3 are a population of interest for intra-abdominal candidiasis studies, with a prevalence of 44%.
- The combination of serum and peritoneal 1.3-beta-d-glucan with the peritonitis score emerged as a potent strategy for effectively excluding intra-abdominal candidiasis and thereby minimizing unnecessary exposure to antifungal agents.

## Introduction

Intra-abdominal candidiasis (IAC) is defined by the detection of *Candida* in peritoneal fluid obtained through direct puncture, intraoperative sampling, or drainage from an intra-abdominal drain inserted in the past 24 h, along with compatible signs and symptoms of intra-abdominal infection [1, 2]. The definitive diagnosis of IAC relies on the isolation of *Candida* species through conventional mycological culture. IAC carries a cumulative incidence of 1.84 cases per 1000 intensive care unit (ICU) admissions [3] and is associated with a mortality rate of up to 60% [4].

IAC is difficult to predict in critically ill patients with intra-abdominal infection and could lead to delayed [4] or excessive use of antifungal treatments [5]. Indeed, conventional culture could take several days to yield results [6]. Considering the worse prognosis of patients with delayed introduction, antifungals are usually introduced before the results of the culture are obtained, based on clinical scores or context. However, none of the current clinical scores are able to identify patients at risk for IAC, leading to antifungal overuse [5, 7]. Unnecessary antifungal exposure has been associated with increased antifungal resistance, including against echinocandins, the first class used for IAC treatment [8].

To optimize the diagnosis of IAC, the measurement of 1.3-beta-d-glucan (BDG) in the serum (sBDG) and peritoneal fluid (pBDG) has gained interest [9–12]. BDG is a crucial constituent of the cell wall of various fungal species, including *Candida*. In IAC, sBDG is associated with a negative predictive value (NPV) ranging from 70 to 90% [13, 14], and two measurements in 48 h are needed. For pBDG, three studies have reported higher concentrations in IAC patients than in non-IAC individuals [10–12]. However, the statistical significance of these differences in pBDG concentrations has not been consistently established. In addition, the actual prevalence of IAC in these studies was < 30%. Thus, the literature does not provide a definitive conclusion regarding the utility of pBDG in confirming or excluding IAC.

Furthermore, all these studies employed the Fungitell^®^ beta-d-glucan assay (FA, Associate of Cape Cod, East Falmouth, Inc., United States of America) for pBDG measurement. The Wako^®^ beta-glucan test (WT, Fujifilm Wako Chemicals Europe, Neuss, Germany) has not been assessed in this context before. The WT has been validated for both serum and plasma matrices. This BDG test is technically less complex to operate than the FA (Fungitell assay) and is simpler to execute and interpret [15]. Therefore, it is plausible that the WT would be more suitable for the medium represented by peritoneal fluid.

The present study sought to identify a high risk of IAC in critically ill patients and to evaluate pBDG measurements using the beta-glucan test from Wako^®^ for the diagnosis of IAC. This study aimed to address some of the limitations of prior research, such as patient population heterogeneity and insufficient confirmed IAC cases, which can introduce bias and ambiguity into the results [16].

## Material and methods

### Study design: setting

**Fig. 1:**
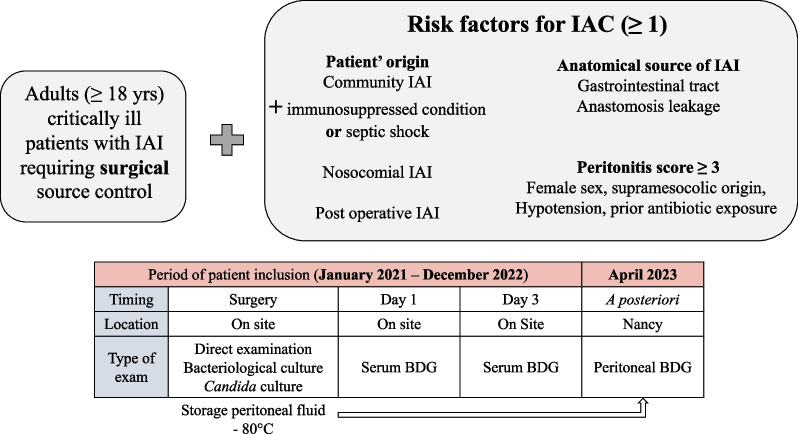
Study design and inclusion criteria. Abbreviations: BDG: 1.3 beta-d-glucan; IAC: intra-abdominal infection; IAI: intra-abdominal infection. Risk factors according to [19–22], Peritonitis score according to [23]

This was a French prospective multicenter noninterventional study conducted at four tertiary teaching hospitals (Dijon, Metz, Nancy and Strasbourg). The protocol for the pBDG2 study has been previously published [17] and summarized in the Fig. 1.

The initial recruitment period spanned from January 1st, 2020, to December 31st, 2021. However, due to the COVID-19 outbreak, this period was subsequently extended by 1 year. The management of patients and their anti-infective strategies were left to the discretion of the attending physicians but were required to adhere to the current guidelines for the management of invasive candidiasis [18, 19].

### Participants

Critically ill adult patients with an intra-abdominal infection that necessitated surgical intervention and risk factors IAC [19–22] were included consecutively (Fig. 1).

The primary focus of the study was the diagnostic assessment of pBDG, and consequently, patient follow-up was limited to their duration of stay in the ICU.

### Gold standard test for the diagnosis of IAC

The definition of IAC relied on positive culture findings of peritoneal fluid collected under sterile conditions for *Candida* species. The assessment of *Candida* growth was conducted using Sabouraud chloramphenicol medium (BioMerieux, Craponne, France) at a temperature of 35 ± 2 °C. Additional species identification was carried out employing mass spectrometry, and the cultures were retained for a period of up to 8 days.

Serum and peritoneal BDG.

Serum (sBDG) concentrations were collected on Day 1 and Day 3 following abdominal surgery, aligning with established expert guidelines [24] (see Additional file 1 for details of the test used**)**.

For pBDG, any residual peritoneal fluid after routine analysis was preserved at each center using BDG-free containers at a temperature of − 20 °C until the conclusion of the recruitment period. Subsequently, all these samples were shipped to the Nancy Center, where BDG measurements using the WT were conducted between May and June 2023.

### Objectives

The primary objective was to estimate the prevalence of IAC in the studied population. The secondary objectives were (1) to compare the pBDG concentrations between patients with and without IAC, (2) to identify the risk factors associated with the development of IAC, (3) to assess the diagnostic accuracy of pBDG for the early detection of IAC, using *Candida* culture as the reference standard, and (4) to assess the diagnostic performances of pBDG, both alone and in combination with sBDG and the peritonitis score, to assess the presence of IAC. sBDG results were considered negative (indicating a low risk of IAC) when levels were less than 80 pg/ml (using FA) or less than 3.3 pg/ml (using WT) on two consecutive measurements, according to the literature [25, 26].

### Data collection

We collected data on demographics, comorbidities, type of intra-abdominal infection, and IAC management from electronic medical records during patients' ICU stays (see Additional file 1 for details). Previous *Candida* colonization was defined as the isolation of *Candida* in cultures obtained from ≥ 2 of the following sources: respiratory tract secretions, stool, skin, wound sites, urines, and drains that have been in place for 24 h or less [1].

### Statistical analysis

According to the literature, the prevalence of IAC in severe intra-abdominal infection is estimated to be between 20 and 40% [10, 27]. The expected prevalence of IAC in our sample was 30%, based on our previous study [10]. The inclusion of 200 patients enabled us to estimate this expected prevalence with an absolute precision of 6.5%.

Descriptive statistics were used to summarize the baseline characteristics of the study population, including counts and percentages for categorical variables and the mean ± standard deviation or median [interquartile range/IQR] for continuous variables, depending on the data distribution. Statistical tests such as the chi-squared or Fisher’s exact tests were employed to compare categorical variables, while the nonparametric Mann‒Whitney U test and Kruskal‒Wallis test were used for continuous variables. The prevalence of IAC was calculated based on *Candida* culture results, along with the 95% confidence interval.

Regarding the secondary objectives, initial sBDG and pBDG concentrations were compared using the Wilcoxon test. Risk factors for IAC were identified through bivariate logistic regression. Factors with a significance threshold of 0.10 were considered candidates in a multivariable regression model, with significance defined as a *P* value of < 0.05 in two-sided tests.

To assess the diagnostic performance of pBDG, a receiver operating characteristic (ROC) curve analysis was conducted, and a new cutoff value was determined, considering the highest Youden index (sensitivity + specificity − 1). For sBDG, analyses were performed based on the test used and previously published thresholds [25]. All statistical analyses were carried out by an independent biostatistician using SAS v9.4 (SAS Institute, Inc., Cary, NC), with a significance level of *P* < 0.05.

## Results

### Demographic and characteristics

**Fig. 2:**
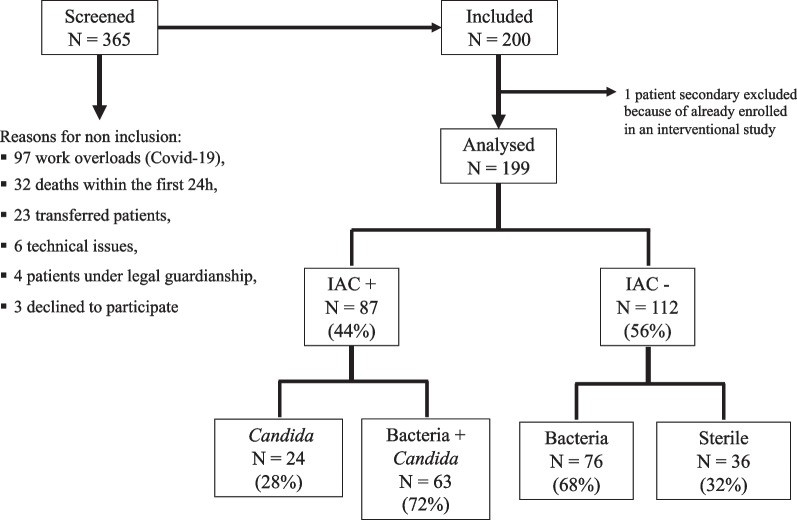
Flow chart of the study

Figure 2 illustrates the study’s flow chart.

Table 1 presents the clinical characteristics of the 199 patients included in the analysis. Patients were predominantly male (63%), with a median age of 66 [54–72] years. Intra-abdominal infections were community-acquired in 27% of cases (n = 54).

**Table 1:** Critically ill patients clinical characteristics

Variables	IAC (n = 87)	No IAC (n = 112)	*P value*
Age (years)	67 [59–72]	64 [53–72]	0.26
Male sex	54 (62)	72 (64)	0.75
Body mass index (kg/m^2^)	26.2 [23.2–32.0]	25.9 [21.6–31.0]	0.38
Knaus score^a^			0.26
A	16 (18)	12 (11)	
B	51 (59)	68 (61)	
C	20 (23)	32 (28)	
McCabe score^b^			0.58
1	47 (54)	53 (47)	
2	34 (39)	52 (46)	
3	6 (7)	7 (6)	
Comorbidities			
Cardiovascular	14 (16)	17 (15)	0.86
COPD	13 (15)	16 (14)	0.40
Chronic renal insufficiency^c^	19 (22)	15 (13)	0.13
Cirrhosis	4 (5)	11 (10)	0.17
Diabetes	17 (19)	29 (26)	0.29
Peptic ulcer	8 (9)	18 (16)	0.15
Malnutrition^d^	60 (69)	65 (58)	0.11
Immunocompromised^e^	34 (39)	43 (38)	0.92
ICU data			
Admission SAPS II score	49 [35–62]	50 [35–60]	0.91
IAC diagnosis SOFA score	6.5 [4–9]	6 [4–9]	0.66
Septic shock (Sepsis 3)	56 (64)	68 (61)	0.60
Norepinephrine infusion	70 (80)	82 (73)	0.19
Invasive mechanical ventilation > 48 h	36 (51)	47 (42)	0.93
Renal replacement therapy	23 (41)	19 (26)	0.08
ICU mortality	14 (16)	13 (12)	0.36
Peritonitis data			
Peritonitis score ≥ 3	54 (62)	52 (46)	**0.03**
Post-operative peritonitis	50 (57)	54 (48)	0.19
Site of origin			0.73
Infra mesocolic	48 (55)	59 (53)	
Supra mesocolic	39 (45)	53 (47)	
Mechanism			0.10
Perforation	47 (54)	65 (59)	
Necrosis	13 (15)	25 (22)	
Anastomosis leakage	27 (31)	21 (19)	
Generalized peritonitis	45 (52)	45 (41)	0.11
Presence of bacteria	63 (72)	76 (68)	0.49
Confounding factors			
Antibiotic > 72 h prior to surgery	41 (47)	30 (27)	**0.003**
Albumin perfusion prior to surgery	15 (17)	13 (12)	0.26
Transfusion prior to surgery	18 (21)	23 (21)	0.98
*Candida* colonisation^f^ prior to surgery	8 (9)	8 (7)	0.60

Results expressed as n (%) and median [IQR]

*COPD* chronic obstructive pulmonary disease, *IAC* intra-abdominal candidiasis, *ICU* intensive care unit, *RRT* renal replacement therapy, *SAPS II* Simplified Acute Physiological Score, *SOFA* Sequential Organ Failure Assessment score

^a^Knaus score [28]: class A (Normal health status)—B (Moderate activity limitation)—C (Severe activity limitation due to chronic disease)—D (Bedridden patient).

^b^McCabe score [29]: class 1 (Nonfatal disease)—2 (Ultimately fatal disease)—3 (Rapidly fatal disease)

^c^Chronic renal insufficiency: defined as an estimated glomerular filtration rate < 60 mL/min/1.73 m^2^

^d^Malnutrition: based on phenotype and etiology criteria from the ESPEN GLIM recommendations [30]

^e^Immunocompromised: active cancer (solid tumour or haematological malignancy), organ transplant or bone marrow transplant, systemic and/or immune disease requiring immunosuppressed therapy, receiving one or more immunosuppressed therapy(ies) more than three months.

^f^*Candida* colonization: isolation of *Candida* in cultures obtained from ≥ 2 of the following: respiratory tract secretions, stool, skin, wound sites, urines, and drains that have been in place ≤ 24 h [1]

Antibiotic exposure for more than 72 h prior to surgery and a peritonitis score ≥ 3 were significantly more common in the IAC group (*P* = 0.003 and *P* = 0.03, respectively). One-third of the cohort was considered immunocompromised, mainly due to active solid tumors (n = 52/77). Two-thirds of the patients were admitted to the ICU for urgent intra-abdominal infection, with secondary peritonitis accounting for 96% of cases (n = 192), originating primarily from the colon (n = 71/36%) and small bowel (n = 67/33%). Surgery was predominantly performed by laparotomy (n = 184/92%). Approximately 64% (n = 123) of cases received antibiotic therapy before surgical incision. Eight patients received antifungal treatment before surgery.

During and after surgery, an antifungal treatment was started for 127 patients (64%) primarily empirically (74% of cases). In the IAC group, 30 patients (35%) received antifungal treatment after *Candida* documentation. Regarding the non-IAC group, 42 patients (37%) received unnecessary antifungal therapy. All patients received an echinocandin as empirical therapy, which was de-escalated to fluconazole in 47% of cases. The overall median duration of antifungal therapy was 8 [8] days in the IAC group versus 5 [3–8] days in the non-IAC group (*P* < 0.0001). The median ICU length of stay was 8 [4–14] days, and the ICU mortality rate was 14% (n = 27).

### Primary objective

The prevalence of IAC was 44% (n = 87/199; 95% CI [37–51]). There was no statistically significant difference in *Candida* risk factors between the two groups except for prior antibiotic exposure longer than 72 h (Table 1). A total of 101 *Candida*-positive species were recovered from the 87 PF samples: 65 *C. albicans*, 19 *C. glabrata*, 7 *C. tropicalis*, 4 *C. kefyr*, 3 *C. krusei*, and 2 *C. parapsilosis*. The IAC was polymicrobial in 72% (n = 63) of cases. The most encountered bacteria were *Escherichia coli* and *Enterococcus faecalis* (see Additional file 1: Table S1 for the whole bacterial documentation). Direct examination for yeast was positive for 34 patients (17%). There were only 3 cases of candidemia (3%).

### Secondary objectives

#### Peritoneal BDG

**Fig. 3:**
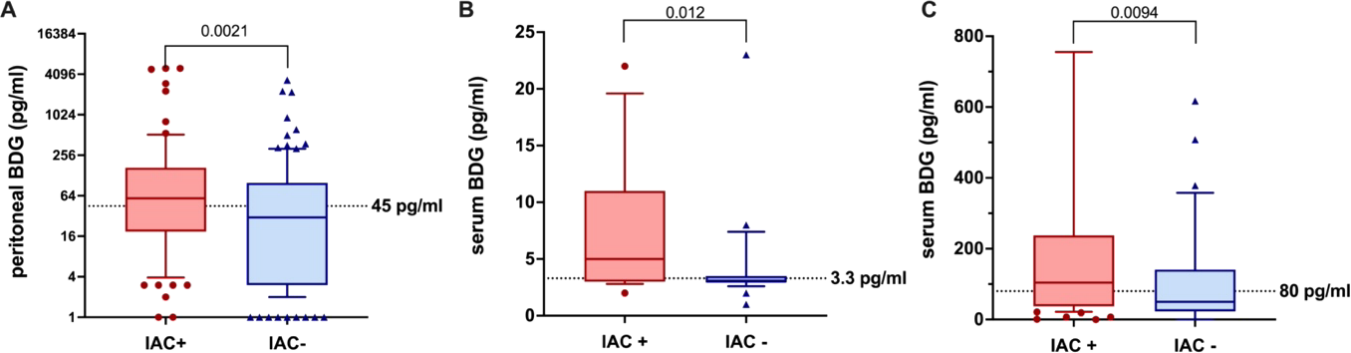
Serum and peritoneal 1.3 beta-d-glucan concentrations in IAC patients versus non-IAC. **A** Box and whiskers with median, 10 and 90% percentile of peritoneal BDG between confirmed IAC and non-IAC patients (Wako^®^ beta-glucan test, Fujifilm Wako Chemicals Europe, Neuss, Germany). Dotted line represents the threshold of 45 pg/ml. **B** Box and whiskers with median, 10 and 90% percentile of serum BDG measured with the Wako^®^ beta-glucan test (Fujifilm Wako Chemicals Europe, Neuss, Germany) at Day 1 between confirmed IAC and non-IAC patients. Dotted line represents the threshold of 3.3 pg/ml (WT). **C** Box and whiskers with median, 10 and 90% percentile of serum BDG measured with the Fungitell^®^ beta-d-glucan assay (FA, Associate of Cape Cod, East Falmouth, Inc., United States of America) at Day 1 between confirmed IAC and non-IAC patients. Dotted line represents the threshold of 80 pg/ml (FA). Abbreviations: IAC: intra-abdominal candidiasis; BDG: 1.3 beta-d-glucan; WT: Wako^®^ beta-glucan test (Fujifilm Wako Chemicals Europe, Neuss, Germany); FA: Fungitell^®^ beta- d-glucan assay (Associate of Cape Cod, East Falmouth, Inc., United States of America)

pBDG results (Fig. 3A) were obtained for 196 PF samples, among which 72 (36%) displayed values higher than 600 pg/ml (the upper limit of the calibration curve). The median pBDG concentration was significantly higher in patients with IAC (448 [107.5–1578.0] pg/ml) than in those without IAC (133 [16.0–831.0] pg/ml) (*P* = 0.0021). The median pBDG levels depending on the results of the peritoneal fluid culture (bacteria, sterile) are provided in aDditional file 1: Fig. S1. Among the risk factors for IAC, after multivariate analysis, a pBDG level ≥ 284 pg/ml and the nosocomial origin of the patient were significantly associated with the presence of IAC (OR 2.5 [1.3–4.5]; *P* = 0.003) and OR 2.1 [1.2–3.9]; *P* = 0.014), respectively (see Additional file 1: Tables S3/S4).

#### Serum BDG

Using the WT (n = 42, Fig. 3B), in IAC patients, the median sBDG concentration was significantly higher than that in non-IAC patients, measuring 5 [3.0–9.0] pg/ml versus 3 [3.0–3.0] pg/ml, *P* = 0.012. Patients with sBDG < 3.3 pg/ml had a significantly lower occurrence of IAC (*P* = 0.004).

Using the FA (n = 140, Fig. 3C), in IAC patients, the median sBDG concentration was significantly higher than that in non-IAC patients, measuring 104 [38.0–211.0] pg/ml versus 50 [23.0–141.0] pg/ml, *P* = 0.0094. Patients with sBDG < 80 pg/ml had a significantly lower occurrence of IAC (*P* = *0.087*).

The Additional file 1: Fig. S2 shows the distribution of sBDG on Day 1 and 3 in both groups.

#### Diagnostic performance

The diagnostic performance of pBDG, sBDG, and the peritonitis score is reported in Table 2. Combining diagnostic tests was the best approach to obtain an NPV of 90% (sBDG measured with the FA) and 100% (sBDG measured with the WT) for ruling out IAC.

**Table 2:** Diagnostic performance of tests used alone and combined considering the *Candida* culture of peritoneal samples

Test	Number of patients above the threshold	Sensitivity	Specificity	Positive predictive value	Negative predictive value
Peritonitis score ≥ 3	106/199	62.1 [51.6–71.5]	53.6 [44.4–62.5]	50.9 [41.6–60.3]	64.5 [54.4–73.5]
Serum BDG (FA) ≥ 80 pg/ml	65/140	59.0 [46.5–70.5]	63.3 [52.3–73.1]	55.4 [43.3–66.8]	66.7 [55.4–76.3]
Serum BDG (WT) ≥ 3.3 pg/ml	18/42	70.6 [46.9–86.7]	76.0 [56.6–88.5]	66.7 [43.7–83.7]	79.2 [59.5–90.8]
Peritoneal BDG (WT) ≥ 45 pg/ml	145/196	89.5 [81.3–94.4]	38.2 [29.6–47.5]	53.1 [45.0–61.0]	82.3 [69.7–90.4]
Peritoneal BDG (WT) ≥ 45 pg/ml or serum BDG (FA) ≥ 80 pg/ml or peritonitis score ≥ 3	130/140	98.5 [92.2–99.7]	11.1 [5.1–18.3]	48.6 [40.4–56.8]	90.0 [59.6–98.2]
Peritoneal BDG (WT) ≥ 45 pg/ml or serum BDG (WT) ≥ 3.3 pg/ml or peritonitis score ≥ 3	35/42	100.0	28.0 [14.3–47.6]	48.6 [33.0–64.4]	100.0

Results expressed as % [95% confidence interval]. The results of serum BDG that were considered was Day 1. Serum BDG with FA test: n = 140 (61 IAC+ /79 IAC−) with WT: n = 42 (17 IAC+ /25 IAC−).

BDG: 1.3 beta-d-glucan; FA: Fungitell^®^ beta-d-glucan assay (Associate of Cape Cod, East Falmouth, Inc., United States of America); WT: Wako^®^ beta-glucan test (Fujifilm Wako Chemicals Europe, Neuss, Germany)

**Fig. 4:**
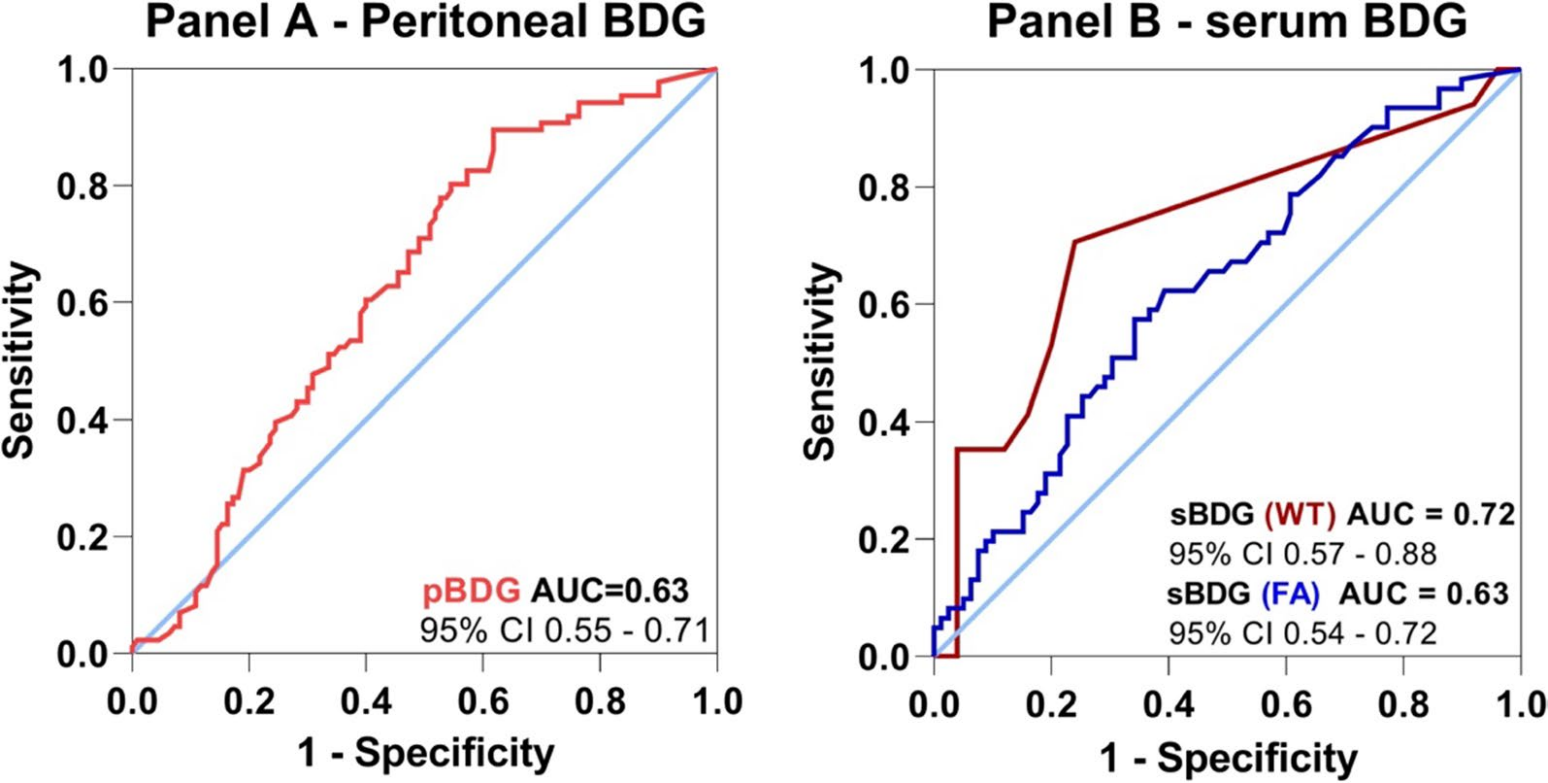
Peritoneal and serum 1.3 beta-d-glucan to rule out intra-abdominal candidiasis in secondary peritonitis. Receiver operating characteristic curve of peritoneal and serum 1.3 beta-d-glucan for identification of intra-abdominal candidiasis (**A**—peritoneal BDG (n = 196)/**B**—serum BDG at Day 1 according to the test used (Wako test^®^ N = 42 and Fungitell^®^ beta-d-glucan assay N = 140). Abbreviations: FA: Fungitell^®^ beta-d-glucan assay (Associate of Cape Cod, East Falmouth, Inc., United States of America); pBDG: peritoneal 1.3 beta-d-glucan; sBDG: serum 1.3 beta-d-glucan; WT: Wako^®^ beta-glucan test (Fujifilm Wako Chemicals Europe, Neuss, Germany)

The ROC curve for the diagnostic performance of pBDG and sBDG in the diagnosis of IAC is shown in Fig. 4. Using a cutoff value of 45 pg/ml (determined by the highest Youden index), the NPV was 82.3%. With this threshold, 9 cases of IAC would have been missed. The time to positivity of the *Candida* culture in these 9 cases was 4 days.

## Discussion

In this large cohort of critically ill patients who had undergone urgent abdominal surgery for intra-abdominal infection, the prevalence of IAC was 44%. The study's findings revealed that a pBDG threshold of 45 pg/ml exhibited an NPV of 82.3% (area under the curve (AUC) of 0.63). When combining peritoneal BDG < 45 pg/ml and low serum BDG with a peritonitis score < 3, the negative predictive value reached an impressive 100%.

No demographic data or *Candida* risk factors emerged as predictors for IAC in our study, consistent with previous research [7]. Only the nosocomial origin was associated with more IAC occurrences. This could be explained by the high rate of postoperative infection and prior antibiotic exposure in the cohort, two recognized risk factors for IAC [20]. Interestingly, we did not observe a higher rate of supramesocolic origin in the IAC group. This could be attributed to a lower prevalence of gastrointestinal origin (16%) and more colorectal origin. De Ruiter et al*.* reported high rates of *Candida* during the initial week of intra-abdominal infection originating from colorectal origin [31], aligning with the timeframe of post-operative peritonitis. Additionally, we noted a balanced distribution of community origin between IAC and non-IAC patients, consistent with previous findings [12, 31, 32]. This underscores the importance of considering community origin when contemplating the initiation of empirical antifungal therapy, particularly in the presence of immunosuppression [19] and/or septic shock [22]. Additionally, because we only included patients with intra-abdominal infection requiring surgery, the prevalence of IAC increased. Indeed, Dupont et al. reported a 30% prevalence of IAC in a population with complicated intra-abdominal infection requiring surgery [11]. In contrast, Nourry et al. included patients with intra-abdominal infection managed by radiology and reported a prevalence of 21% [12]. Recent literature consistently supports the idea of focusing on selected ICU populations when evaluating the diagnostic performance of biomarkers for IAC such as pBDG [1, 9, 21]. Consequently, studying ICU populations that necessitate surgical source control emerges as a promising approach for assessing biomarkers for IAC.

Two studies with an IAC prevalence of 21% reported promising results when measuring pBDG in critically ill patients to rule out IAC [10, 12]. In the first retrospective study including 33 nosocomial secondary peritonitis cases, the authors reported a 98% NPV [10]. Recently, Nourry et al. conducted a prospective study with 113 patients and reported a 100% NPV [12]. In our study using the WT, the NPV of pBDG was notably lower, at 82.3%. First, as the NPV depends on the prevalence of the disease, its value decreases with increasing prevalence. Additionally, it is important to consider that in previous studies, the actual number of patients with IAC whose pBDG concentrations fell below the obtained threshold was low, at 3 patients in the first study and 12 patients in the second study. Furthermore, in the study by Nourry et al., the authors acknowledged that 21 samples had been exposed to antifungal treatment, which could lead to negative fungal culture results and/or reduced yeast quantities [12]. In our study, using pBDG alone with a threshold of 42 pg/ml would have led to nine missed cases. Notably, the mean time to positivity of the *Candida* culture in these nine cases was four days, suggesting a low inoculum. Thus, the quantity of yeast present might have influenced the diagnostic performance of pBDG.

The pBDG levels were higher in cases of polymicrobial IAC and lower in negative samples, which is consistent with previous findings [10, 12]. Similarly to previous study [4, 31], we reported a high bacterial documentation of 72%. Bacteria, especially gram-negative and enterococci are known to be associated with false-positive results for BDG [33], which could explain the high rate of false-positive peritoneal BDG results observed in our study. With a threshold of ≥ 45 pg/ml, 68 patients would have received unnecessary antifungal treatment.

Thus, we reaffirmed the limited positive predictive performance of both peritoneal and serum BDG. In the ICU setting, numerous confounding factors for sBDG exist, including antibiotics, albumin infusion, and transfusions, among others, which are known to be associated with false-positive results [33]. Additionally, Szyszkowitz et al*.* highlighted increased levels of sBDG in the peri- and post-operative period, diminishing the significance of its positive value [34]. Conversely, the risk of false-negative values is less likely to occur, particularly in the peritoneal fluid. False negatives are typically caused by a low inoculum or prior exposure to antifungals [35]. In the case of IAC, clinical studies have reported a low rate of antifungal initiation before surgical incision [5], and the peritoneal diffusion of antifungals has been demonstrated to be low (approximately 30%) [36].

The results of our study regarding sBDG differed from previously published data [11]. In the study by Dupont et al., the sBDG determined with the FA showed an AUC of 0.52, *P* = 0.77, indicating poor diagnostic performance. In our study, both tests demonstrated significantly lower levels of sBDG in non-IAC cases, with reported AUCs of 0.63 and 0.72 for the FA and WT, respectively. Previously, sensitivity values of approximately 70% have been documented for both tests in ICU patients with IAC and no concurrent candidemia [25]. Interestingly, the diagnostic performance of the WT alone and combined with the other markers was superior to that of the FA in our study. Additionally, our study confirmed the superior sensitivity of the WT when using a lower cutoff of 3.3 pg/ml compared to 7 pg/ml, as demonstrated previously in critically ill patients with noncandidemic IAC [25].

While our study has highlighted the limitations of using serum and pBDG and the Peritonitis score as standalone markers for initiating antifungal therapy, it has also emphasized the importance of combining tests to rule out IAC [9, 37]. Specifically, the combination of low pBDG and sBDG with a peritonitis score < 3 demonstrated a sensitivity and NPV of 100% and could be used for discontinuing unnecessary treatment in patients with IAC. In our study, we corroborated the limited performance of the peritonitis score when used on its own [11, 12, 38]. However, its combination with BDG demonstrated the potential to enhance diagnostic efficacy (Additional file 1: Table S6). This assessment tool is not only easy-to-use at the bedside but also cost-effective and globally accessible. Furthermore, the current BDG tests (WT or Fungitell STAT assay^®^) permit individual patient testing with a swift turnaround time (less than 90 min) [15]. Consequently, an algorithm grounded in the peritonitis score and individual BDG tests could swiftly exclude IAC within two days, averting unnecessary antifungal exposure linked to escalating antifungal resistance and elevated costs [39]. It is noteworthy that this timeframe necessitates further clinical validation in real-life ICU conditions.

The major strengths of our study include its sample size, multicenter nature, and high number of consecutively included patients with pBDG results, allowing for a high prevalence of IAC. Certainly, there are some limitations to consider. Firstly, there is a risk of misclassification between IAC and non-IAC due to the sensitivity of the peritoneal culture. However, all participating centers followed a local protocol that recommended direct inoculation of the peritoneal sample into a favorable culture medium and storage of all cultures for up to 8 days to detect delayed positivity, which helps mitigate this risk to some extent. Secondly, the COVID-19 outbreak affected our study, and we had to extend the inclusion period. However, the management of IAC remained consistent during this time, which should minimize any potential impact on our results. Thirdly, the peritonitis score was the exclusive scoring system employed in this non-interventional study, aligning with the standard practice across all participating centers and in accordance with the French guidelines [19]. Consequently, the assessment of alternative *Candida* risk factor scores was precluded due to the prevailing routine in the involved centers.

Last, our study did not evaluate patient survival but rather focused on surrogate markers to optimize the detection of *Candida* in peritoneal fluid. The role of *Candida* as a true pathogen in IAC is still debated [40, 41], and the effectiveness of antifungal treatment in IAC remains inconclusive in previous studies [4, 42, 43].

## Conclusion

In critically ill patients with secondary peritonitis, the IAC prevalence was 44%. Our study advocates for a comprehensive strategy involving a peritoneal BDG measurement below 45 pg/ml (Wako^®^ beta-glucan test), coupled with a Peritonitis score below 3 and low serum BDG levels on day 1, to effectively exclude *Candida* infection within a span of two days. Nonetheless, further clinical validation in ICU real-life is warranted.

### Supplementary Information

**Additional file 1:** Combination of serum and peritoneal 1.3-beta-D-glucan can rule out intra-abdominal candidiasis in surgical critically ill patients: A multicenter prospective study. File format: pdf. Including further details on Results with seven tables (Table S1 “Bacteriology data”, S2 “Antibiotic therapy”, S3 “Risk factors for intra-abdominal candidiasis (univariate analysis)”, S4 “Risk factors for intra-abdominal candidiasis (multivariate analysis)”, S5 “Diagnostic performance of different serum and peritoneal beta-d-glucan threshold considering the Candida culture of peritoneal samples”, S6 “Influence of the peritonitis score on the diagnostic performance of serum and peritoneal 1.3 beta-d-glucan”, S7 “Peritoneal 1.3 beta-d-glucan results depending on the community and nosocomial origin of intra-abdominal infection”) and two figures (Figure S1 “Peritoneal 1.3 beta-d-glucan concentrations according to the culture of peritoneal fluid sample”, S2 “Distribution of serum BDG at Day 1 and Day 3 according to the test used”), on the Participating centers, on Methods, and the checklists from STROBE and STARD reporting guidelines.

## Data Availability

The datasets used and/or analyzed during the current study are available from the corresponding author upon reasonable request.

## Abbreviations

AUC: Area under the curve
BDG: 1.3 Beta-d-glucan
FA: Fungitell^®^ beta-d-glucan assay (Associate of Cape Cod, East Falmouth, Inc., USA)
IAC: Intra-abdominal candidiasis
ICU: Intensive care unit
NPV: Negative predictive value
pBDG: Peritoneal 1.3 beta-d-glucan
pBDG2: Prospective evaluation of the interest of 1.3-beta-d-glucan in the peritoneal fluid for the diagnosis of intra-abdominal candidiasis in critically ill patients
ROC: Receiving operating characteristic
sBDG: Serum 1.3 beta-d-glucan
WT: Wako^®^ beta-glucan test (Fujifilm Wako Chemicals Europe, Neuss, Germany)
