# Supplementary material for: Combination of serum and peritoneal 1.3-beta-d-glucan can rule out intra-abdominal candidiasis in surgical critically ill patients: a multicenter prospective study
Source: Crit Care. 2023 Nov 30;27:470. doi: 10.1186/s13054-023-04761-7 (PMC10691030; doi:10.1186/s13054-023-04761-7)
Supplement: Supplementary file 1 — Additional file 1: Combination of serum and peritoneal 1.3-beta-D-glucan can rule out intra-abdominal candidiasis in surgical critically ill patients: A multicenter prospective study. File format: pdf. Including further details on Results with seven tables (Table S1 “Bacteriology data”, S2 “Antibiotic therapy”, S3 “Risk factors for intra-abdominal candidiasis (univariate analysis)”, S4 “Risk factors for intra-abdominal candidiasis (multivariate analysis)”, S5 “Diagnostic performance of different serum and peritoneal beta-d-glucan threshold considering the Candida culture of peritoneal samples”, S6 “Influence of the peritonitis score on the diagnostic performance of serum and peritoneal 1.3 beta-d-glucan”, S7 “Peritoneal 1.3 beta-d-glucan results depending on the community and nosocomial origin of intra-abdominal infection”) and two figures (Figure S1 “Peritoneal 1.3 beta-d-glucan concentrations according to the culture of peritoneal fluid sample”, S2 “Distribution of serum BDG at Day 1 and Day 3 according to the test used”), on the Participating centers, on Methods, and the checklists from STROBE and STARD reporting guidelines. [file 13054_2023_4761_MOESM1_ESM.docx]

**Additional file 1**

Further details on **Results**

1. **Table S1: Bacteriology data**

|  | N (%) |
| --- | --- |
| Positive direct examination | 93 (47) |
| Positive bacterial culture | 139 (70) |
| Gram-negative aerobes:  *E. coli*  *Klebsiella spp.*  *Citrobacter spp.*  *Morganella spp.*  *Serratia spp.*  *Hafnia spp.*  *Enterobacter spp.*  *Proteus spp.*  *Providencia spp.*  *P. aeruginosa*  *A. baumanii* | 67  16  10  7  1  2  17  8  1  14  1 |
| Gram-positive cocci:  *S. aureus*  *S. epidermidis*  *E. faecalis*  *E. faecium*  *Streptococcus spp.* | 8  6  52  24  19 |
| Anaerobes:  *Bacteroides spp.*  *Clostridium spp.* | 43  12 |
| Bacteraemia | 23 (11) |

1. **Table S2: Antibiotic therapy**

|  | N (%) |
| --- | --- |
| Antibiotic prior to surgery | 128 (64) |
| Empirical antibiotic therapy:  Beta-lactam  Aminoglycoside  Metronidazole  Vancomycin  Linezolid | 186 (94)  173  60  24  20  53 |
| Directed therapy:  Beta-lactam  Aminoglycoside  Metronidazole  Vancomycin  Linezolid | 131 (66)  128  3  8  8  16 |
| Duration (days) | 1. [7-8] |

1. **Table S3: Risk factors for intra-abdominal candidiasis (univariate analysis)**

| Variable | N | IAC + |  | | |
| --- | --- | --- | --- | --- | --- |
|  | | | OR | 95% CI | *P* |
| Peritoneal BDG  < 284 pg/ml  ≥ 284 pg/ml | 94  102 | 32 54 | 1  2.2 | [1.2 – 3.9] | **0.0082** |
| Female sex | 73 | 33 | 1.1 | [0.6 – 2.0] | 0.7473 |
| Malnutrition | 125 | 60 | 1.6 | [0.9 – 2.9] | 0.1146 |
| Immunocompromised | 77 | 34 | 1 | [0.6 – 1.8] | 0.9213 |
| Prior *Candida* colonisation | 16 | 8 | 1.3 | [0.5 – 3.7] | 0.5983 |
| Septic shock | 124 | 56 | 1.2 | [0.7 – 2.1] | 0.5979 |
| Nosocomial origin vs septic shock and community-acquired | 102 | 51 | 4 | [0.8 – 19.8] | **0.0964** |
| Post-operative peritonitis | 104 | 50 | 1.5 | [0.8 – 2.6] | 0.1954 |
| Infra mesocolic origin | 107 | 48 | 1.1 | [0.6 – 1.9] | 0.7265 |
| Generalized peritonitis | 90 | 45 | 1.6 | [0.9 – 2.8] | 0.1123 |

1. **Table S4: Risk factors for intra-abdominal candidiasis (multivariate analysis)**

| Variable | N | IAC + |  | | |
| --- | --- | --- | --- | --- | --- |
|  | | | OR | 95% CI | *P* |
| Peritoneal BDG  < 284 pg/ml  ≥ 284 pg/ml | 94  102 | 32 54 | 1  2.5 | [1.3 – 4.5] | **0.003** |
| Nosocomial origin vs septic shock and community-acquired | 102 | 51 | 2.1 | [1.2 – 3.9] | **0.014** |

1. **Table S5: Diagnostic performance of different serum and peritoneal beta-d-glucan threshold considering the *Candida* culture of peritoneal samples**

| Test | Number of patients above the threshold | Sensitivity | Specificity | Positive Predictive Value | Negative Predictive Value |
| --- | --- | --- | --- | --- | --- |
| Serum BDG (WT)  ≥ 3.3 pg/ml | 18/42 | 70.6  [46.9-86.7] | 76.0  [56.6-88.5] | 66.7  [43.7-83.7] | 79.2  [59.5-90.8] |
| Serum BDG (WT)  ≥ 7 pg/ml | 9/42 | 35.3  [17.3-58.7] | 88.0  [70.0-95.8] | 66.7  [35.4-87.9] | 66.7  [49.6-80.2] |
| Peritoneal BDG (WT)  ≥ 10 pg/ml | 171/196 | 94.2  [87.1-97.5] | 18.2  [12.1-26.4] | 47.4  [40.0-54.8] | 80.0  [60.1-91.1] |

Results expressed as % [95% confidence interval]. The results of serum BDG that were considered was Day 1. Serum BDG with FA test: n = 140 (61 IAC+ / 79 IAC -) with WT: n = 42 (17 IAC+ / 25 IAC -).

**Abbreviations:** BDG: 1.3 beta-d-glucan; FA: Fungitell® beta-D-glucan assay (Associate of Cape Cod, East Falmouth, Inc., United States of America); WT: Wako® beta-glucan test (Fujifilm Wako Chemicals Europe, Neuss, Germany).

1. **Table S6: Influence of the peritonitis score on the diagnostic performance of serum and peritoneal 1.3 beta-d-glucan**

| Test | Number of patients above the threshold | Sensitivity | Specificity | Positive Predictive Value | Negative Predictive Value |
| --- | --- | --- | --- | --- | --- |
| Peritoneal BDG (WT) and serum BDG (FA) | | | | | |
| Peritoneal BDG (WT)  ≥ 45 pg/ml  or Serum BDG (FA)  ≥ 80 pg/ml | 129/148 | 94.5  [86.0-97.7] | 19.0  [11.9-29.0] | 50.4  [41.9-59.9] | 78.9  [56.7-91.5] |
| Peritoneal BDG (WT)  ≥ 45 pg/ml  or Serum BDG (FA)  ≥ 80 pg/ml  or Peritonitis score ≥ 3 | 130/140 | **98.5**  [92.2-99.7] | 11.1  [5.1-18.3] | 48.6  [40.4-56.8] | **90.0**  [59.6-98.2] |
| Peritoneal BDG (WT) and serum BDG (WT) | | | | | |
| Peritoneal BDG (WT)  ≥ 45 pg/ml  or Serum BDG (WT) | 32/42 | 100.0 | 40.0  [23.4-59.3] | 53.1  [36.4-69.1] | 100.0 |
| Peritoneal BDG (WT)  ≥ 45 pg/ml  or Serum BDG (WT)  ≥ 3.3 pg/ml  or Peritonitis score ≥ 3 | 35/42 | 100.0 | 28.0  [14.3-47.6] | 48.6  [33.0-64.4] | 100.0 |

Results expressed as % [95% confidence interval]. The results of serum BDG that were considered was Day 1. Serum BDG with FA test: n = 140 (61 IAC+ / 79 IAC -) with WT: n = 42 (17 IAC+ / 25 IAC -).

**Abbreviations:** BDG: 1.3 beta-d-glucan; FA: Fungitell® beta-D-glucan assay (Associate of Cape Cod, East Falmouth, Inc., United States of America); WT: Wako® beta-glucan test (Fujifilm Wako Chemicals Europe, Neuss, Germany).

1. **Table S7: Peritoneal 1.3 beta-d-glucan results depending on the community and nosocomial origin of intra-abdominal infection**

|  | IAC | No IAC | *P* |
| --- | --- | --- | --- |
| Community IAI | 215.7 [180.2-251.2] | 95.5[9.6-3037.0] | < 0.05 |
| Nosocomial IAI | 447.7 [91.1-1578.0] | 28.5[6.7-283.8] |  |

Abbreviations: IAC: intra-abdominal candidiasis; IAI: intra-abdominal infection.

Peritoneal BDG (pg/ml) measured using the Wako® beta-glucan test (Fujifilm Wako Chemicals Europe, Neuss, Germany), results expressed as median, IQR.

1. **Figure S1: Peritoneal 1.3 beta-d-glucan concentrations according to the culture of peritoneal fluid sample**


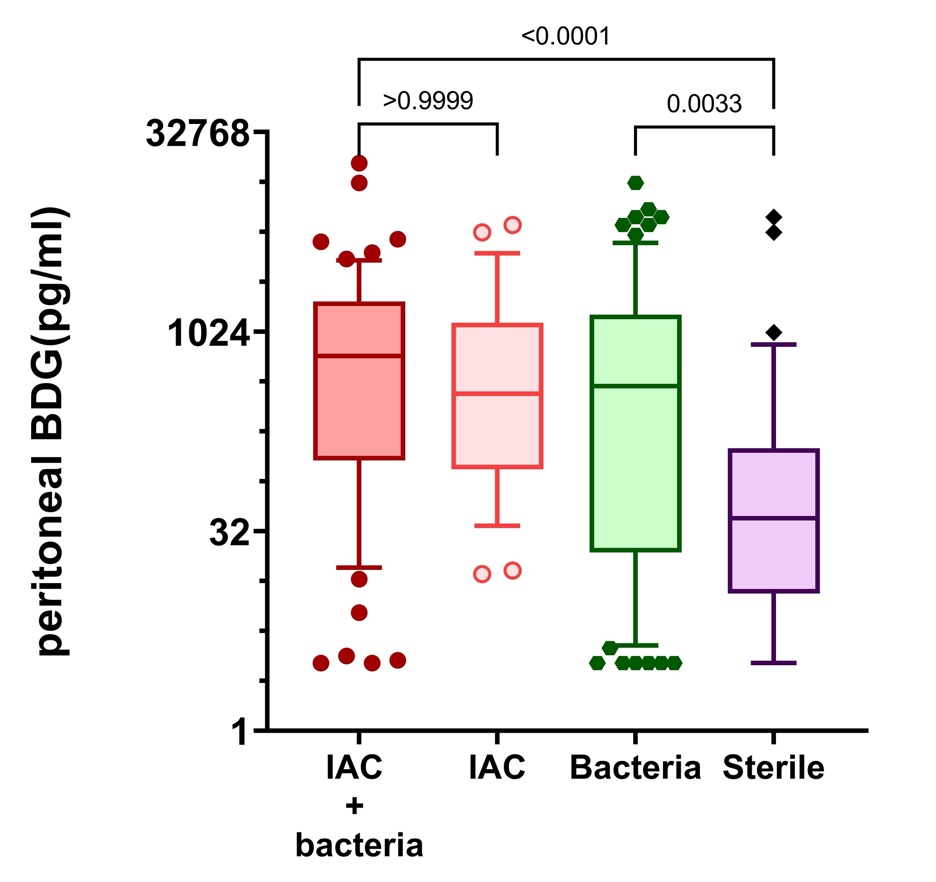


Box and whisker of the median, 10 and 90 percentile confidence of peritoneal 1.3 beta-d-glucan measured with the Wako® beta-glucan test (Fujifilm Wako Chemicals Europe, Neuss, Germany) depending on the results of the peritoneal fluid culture. Comparison between groups was made using Kruskal-Wallis test.

|  | IAC + bacteria | IAC | Bacteria | Sterile |
| --- | --- | --- | --- | --- |
| pBDG (pg/ml) | **669.6** [16.9-3518.0] | **348.6** [35.1-3988.0] | **396.4** [4.4-4771.0] | **40.1** [3.2-817.5] |

Table expressed results as median / 10% and 90% percentile.

1. **Figure S2: Distribution of serum BDG at Day 1 and Day 3 according to the test used**


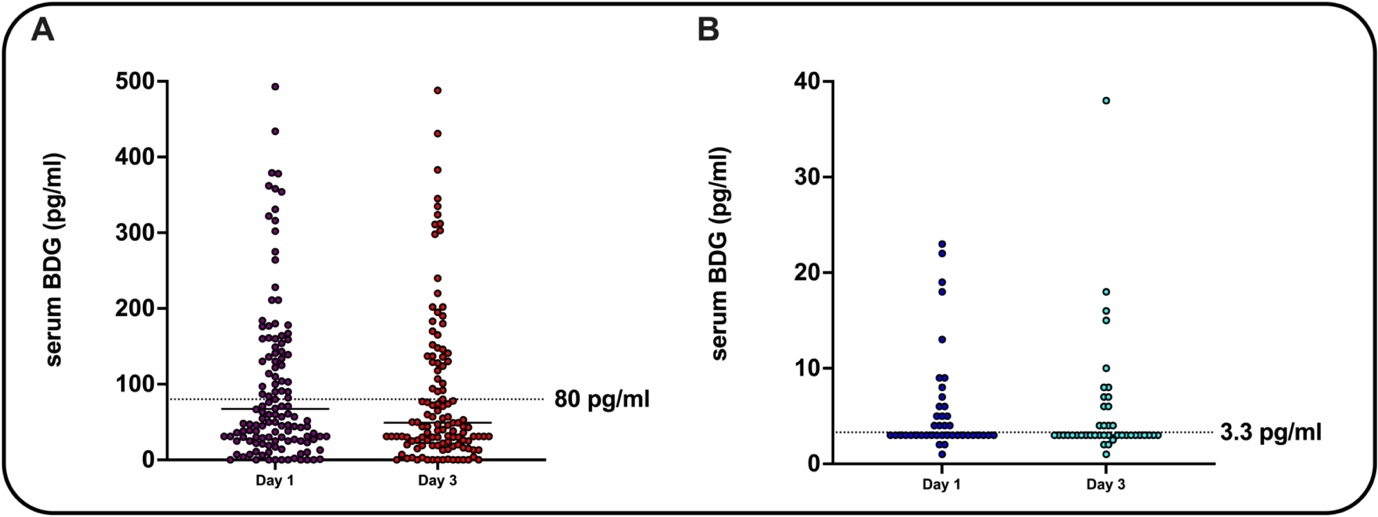


Abbreviations: BDG: 1.3 beta-d-glucan.

Panel A: serum 1.3 beta-d-glucan measured at Day 1 and Day 3 using the Fungitell® beta-D-glucan assay (Associate of Cape Cod, East Falmouth, Inc., United States of America). N=140/157 at Day 1 and 129/157 at Day 3.

Panel B: serum 1.3 beta-d-glucan measured at Day 1 and Day 3 using the Wako® beta-glucan test (Fujifilm Wako Chemicals Europe, Neuss, Germany). N=42/42 at Day 1 and Day 3.

Further details on **Participating centers** and **Methods**

1. **Participating centers**

- Center 1: Dijon

Investigators: Dr NGUYEN Maxime, Pr BOUHEMAD Belaïd.

Affiliation: Department of Anesthesiology and Intensive Care, Dijon University Hospital, 21000 Dijon, France.

Number of included patients: 24.

BDG test used for serum BDG: Fungitell® beta-D-glucan assay (Associate of Cape Cod, East Falmouth, Inc., United States of America).

- Center 2: Metz

Investigators: Dr LOUIS Guillaume, Dr RIVIERE Jeremie, Dr Laithier François-Xavier

Affiliation: Intensive Care Unit, Metz-Thionville Regional Hospital, Mercy Hospital, 57085 Metz, France.

Number of included patients: 17.

BDG test used for serum BDG: the measure is externalised to Nancy so the Fungitell® beta-D-glucan assay (Associate of Cape Cod, East Falmouth, Inc., United States of America) was used until January 2022 and then the Wako® beta-glucan test (Fujifilm Wako Chemicals Europe, Neuss, Germany).

- Center 3: Nancy

Investigators: Dr NOVY Emmanuel, Dr BIRCKENER Julien

Affiliation: Anesthesiology, Critical Care and Perioperative Medicine, University Hospital of Nancy-Brabois, 54500 Vandoeuvre-Lès-Nancy, France.

Number of included patients: 139.

BDG test used for serum BDG: the Fungitell® beta-D-glucan assay (Associate of Cape Cod, East Falmouth, Inc., United States of America) was used until January 2022 and then the Wako® beta-glucan test (Fujifilm Wako Chemicals Europe, Neuss, Germany). This change was not planned at the start of the study as the recruitment period was expected to end in December 2021.

- Center 4: Strasbourg

Investigators: Dr ARFEUILLE Gaëlle, Pr POTTECHER Julien.

Affiliation: Anesthesiology, Critical Care and Perioperative Medicine, Strasbourg University Hospital, 67098 Strasbourg, France

Number of included patients: 19.

BDG test used for serum BDG: Fungitell® beta-D-glucan assay (Associate of Cape Cod, East Falmouth, Inc., United States of America).

1. **Measure of peritoneal BDG.**

The measure of all samples was performed at the laboratory of mycology of the Nancy center. Each sample was measured in duplicate. If the result was above the upper limit of the manufacturer, we made the decision to retain the entire value of peritoneal BDG, rather than attempting to dilute the sample, as dilution could potentially alter the results. This decision was taken by all the mycologists involved in the study and in accordance with Wako®. Seventy-two samples were above the upper limit.

1. **Collected variables.**

These variables included: age, sex, body mass index, comorbidities including immune status, Knauss and McCabe scores at admission, *Candida* risk factors and surgery data, BDG test confounders. The supplementary variables collected were simplified acute physiological score (SAPS II) at admission; sequential organ failure assessment (SOFA) score at admission and at diagnosis of peritonitis, life-support therapies and the ICU mortality rate. Microbiological data (including time to positivity for the fungal culture), types of antibiotics and types of antifungals used were also collected. Empirical antifungal therapy was defined as antifungal therapy started before *Candida* isolation. Introduction of an antifungal treatment after positive for yeast direct examination and/or positive mycological culture was considered as documented antifungal therapy.

Evaluated 1.3-beta-d-glucan test confounders were: beta-lactam exposure, human albumin administration, red blood cell transfusion.

Immunocompromised was defined as followed: having an active cancer (solid tumor or hematological malignancy), being transplanted (bone marrow transplant or solid organ transplant), having systemic and/or immune disease requiring immunosuppressed therapy, receiving one or more immunosuppressed therapy(ies) more than three months.

Type of immunosuppression among included patients:

- Solid organ transplant or Bone Marrow Transplant: 10/77 (13)
- Solid tumor: 52/77 (68)
- Hematological malignancy: 9/77 (12)
- Receiving any immunosuppressive therapy more than 3 months: 19 (25)
- Systemic auto-immune disease: 12/77 (15)

1. **Surgical data**

Type of surgery:

- Laparotomy (n = 184 / 93%)
- Laparoscopy (n = 15 / 7%) – 7 required a switch for laparotomy

Number of post-operative surgical drains: 2 ± 1

**STROBE Statement**

|  | Item No | Recommendation | Page No |
| --- | --- | --- | --- |
| **Title and abstract** | 1 | (*a*) Indicate the study’s design with a commonly used term in the title or the abstract | 1 |
|  |  | (*b*) Provide in the abstract an informative and balanced summary of what was done and what was found | 2 |
| Introduction | | | |
| Background/rationale | 2 | Explain the scientific background and rationale for the investigation being reported | 5-6 |
| Objectives | 3 | State specific objectives, including any prespecified hypotheses | 5-6 |
| Methods | | | |
| Study design | 4 | Present key elements of study design early in the paper | 6 |
| Setting | 5 | Describe the setting, locations, and relevant dates, including periods of recruitment, exposure, follow-up, and data collection | 6-7 |
| Participants | 6 | (*a*) Give the eligibility criteria, and the sources and methods of case ascertainment and control selection. Give the rationale for the choice of cases and controls | 7 (+ Figure 1) |
|  |  | (*b*) For matched studies, give matching criteria and the number of controls per case | NA |
| Variables | 7 | Clearly define all outcomes, exposures, predictors, potential confounders, and effect modifiers. Give diagnostic criteria, if applicable | 8-9  + supplementary material P9 |
| Data sources/ measurement | 8* | For each variable of interest, give sources of data and details of methods of assessment (measurement). Describe comparability of assessment methods if there is more than one group | 9 |
| Bias | 9 | Describe any efforts to address potential sources of bias | 8-9 |
| Study size | 10 | Explain how the study size was arrived at | 9 |
| Quantitative variables | 11 | Explain how quantitative variables were handled in the analyses. If applicable, describe which groupings were chosen and why | 9 |
| Statistical methods | 12 | (*a*) Describe all statistical methods, including those used to control for confounding | 9-10 |
|  |  | (*b*) Describe any methods used to examine subgroups and interactions | 9-10 |
|  |  | (*c*) Explain how missing data were addressed |  |
|  |  | (*d*) If applicable, explain how matching of cases and controls was addressed |  |
|  |  | (*e*) Describe any sensitivity analyses |  |
| Results | | | |
| Participants | 13* | (a) Report numbers of individuals at each stage of study—eg numbers potentially eligible, examined for eligibility, confirmed eligible, included in the study, completing follow-up, and analysed | 11 (Figure 2) |
|  |  | (b) Give reasons for non-participation at each stage | Figure 2 |
|  |  | (c) Consider use of a flow diagram | Figure 2 |
| Descriptive data | 14* | (a) Give characteristics of study participants (eg demographic, clinical, social) and information on exposures and potential confounders | 11 + table 1 |
|  |  | (b) Indicate number of participants with missing data for each variable of interest | 11-18 |
| Outcome data | 15* | Report numbers in each exposure category, or summary measures of exposure | 11-18 |

| Main results | | 16 | (*a*) Give unadjusted estimates and, if applicable, confounder-adjusted estimates and their precision (eg, 95% confidence interval). Make clear which confounders were adjusted for and why they were included | 11-18 |
| --- | --- | --- | --- | --- |
|  |  |  | (*b*) Report category boundaries when continuous variables were categorized | 11-18 |
|  |  |  | (*c*) If relevant, consider translating estimates of relative risk into absolute risk for a meaningful time period |  |
| Other analyses | 17 | Report other analyses done—eg analyses of subgroups and interactions, and sensitivity analyses | | 11-18 |
| Discussion | | | | |
| Key results | 18 | Summarise key results with reference to study objectives | | 19 |
| Limitations | 19 | Discuss limitations of the study, taking into account sources of potential bias or imprecision. Discuss both direction and magnitude of any potential bias | | 22 |
| Interpretation | 20 | Give a cautious overall interpretation of results considering objectives, limitations, multiplicity of analyses, results from similar studies, and other relevant evidence | | 20-22 |
| Generalisability | 21 | Discuss the generalisability (external validity) of the study results | | 20 |
| Other information | | | | |
| Funding | 22 | Give the source of funding and the role of the funders for the present study and, if applicable, for the original study on which the present article is based | | 25 |

*Give information separately for cases and controls.

**Note:** An Explanation and Elaboration article discusses each checklist item and gives methodological background and published examples of transparent reporting. The STROBE checklist is best used in conjunction with this article (freely available on the Web sites of PLoS Medicine at http://www.plosmedicine.org/, Annals of Internal Medicine at http://www.annals.org/, and Epidemiology at http://www.epidem.com/). Information on the STROBE Initiative is available at <http://www.strobe-statement.org>.

**STARD 2015 Statement**

|  | **Section & Topic** | **No** | **Item** | **Reported on page #** |
| --- | --- | --- | --- | --- |
|  |  |  |  |  |
|  | **TITLE OR ABSTRACT** |  |  |  |
|  |  | **1** | Identification as a study of diagnostic accuracy using at least one measure of accuracy  (such as sensitivity, specificity, predictive values, or AUC) | 2 |
|  | **ABSTRACT** |  |  |  |
|  |  | **2** | Structured summary of study design, methods, results, and conclusions  (for specific guidance, see STARD for Abstracts) | 2 |
|  | **INTRODUCTION** |  |  |  |
|  |  | **3** | Scientific and clinical background, including the intended use and clinical role of the index test | 5-6 |
|  |  | **4** | Study objectives and hypotheses | 6 |
|  | **METHODS** |  |  |  |
|  | *Study design* | **5** | Whether data collection was planned before the index test and reference standard  were performed (prospective study) or after (retrospective study) | 6 |
|  | *Participants* | **6** | Eligibility criteria | 6 |
|  |  | **7** | On what basis potentially eligible participants were identified  (such as symptoms, results from previous tests, inclusion in registry) | 7 + Figure 1 |
|  |  | **8** | Where and when potentially eligible participants were identified (setting, location and dates) | 7 |
|  |  | **9** | Whether participants formed a consecutive, random or convenience series | 7 |
|  | *Test methods* | **10a** | Index test, in sufficient detail to allow replication | 7 |
|  |  | **10b** | Reference standard, in sufficient detail to allow replication | 7 |
|  |  | **11** | Rationale for choosing the reference standard (if alternatives exist) | NA |
|  |  | **12a** | Definition of and rationale for test positivity cut-offs or result categories  of the index test, distinguishing pre-specified from exploratory | 8 |
|  |  | **12b** | Definition of and rationale for test positivity cut-offs or result categories  of the reference standard, distinguishing pre-specified from exploratory | 8 |
|  |  | **13a** | Whether clinical information and reference standard results were available  to the performers/readers of the index test | 8 |
|  |  | **13b** | Whether clinical information and index test results were available  to the assessors of the reference standard | 8 |
|  | *Analysis* | **14** | Methods for estimating or comparing measures of diagnostic accuracy | 9 |
|  |  | **15** | How indeterminate index test or reference standard results were handled | 9 |
|  |  | **16** | How missing data on the index test and reference standard were handled | 9 |
|  |  | **17** | Any analyses of variability in diagnostic accuracy, distinguishing pre-specified from exploratory | 9-10 |
|  |  | **18** | Intended sample size and how it was determined | 9 |
|  | **RESULTS** |  |  |  |
|  | *Participants* | **19** | Flow of participants, using a diagram | 11 + Figure 2 |
|  |  | **20** | Baseline demographic and clinical characteristics of participants | 11-14 + Table 1 |
|  |  | **21a** | Distribution of severity of disease in those with the target condition | 11 + Table 1 |
|  |  | **21b** | Distribution of alternative diagnoses in those without the target condition | NA |
|  |  | **22** | Time interval and any clinical interventions between index test and reference standard | 9-10 |
|  | *Test results* | **23** | Cross tabulation of the index test results (or their distribution)  by the results of the reference standard | Table 2 |
|  |  | **24** | Estimates of diagnostic accuracy and their precision (such as 95% confidence intervals) | Table 2 |
|  |  | **25** | Any adverse events from performing the index test or the reference standard | NA |
|  | **DISCUSSION** |  |  |  |
|  |  | **26** | Study limitations, including sources of potential bias, statistical uncertainty, and generalisability | 19-22 |
|  |  | **27** | Implications for practice, including the intended use and clinical role of the index test | 21-22 |
|  | **OTHER INFORMATION** |  |  |  |
|  |  | **28** | Registration number and name of registry | 24 |
|  |  | **29** | Where the full study protocol can be accessed | 6 + 24 |
|  |  | **30** | Sources of funding and other support; role of funders | 25 |
|  |  |  |  |  |
